# Paralysis Following Labor

**Published:** 1879-11

**Authors:** E. A. Hall

**Affiliations:** Odebolt, Iowa


### Article VIII.

*Paralysis Following Labor.*

I was summoned to attend Mrs. F—— in her third confinement. Upon my arrival at her home at about midnight, I found her in the first stage of labor. The first pain was noticed at about ten o'clock — after she had retired for the night. I made a digital examination, and found the os dilated to about the size of a silver quarter, and very soft and moist. The vagina was very moist, and completely relaxed; the vertex R. O. P. presenting. In fact, everything promised well. In answer to her questions, I told her that she might expect a speedy termination of her labor. Dilatation of the os continued to advance rapidly, and it was noticed that true labor pains were taking place. In her previous labors she informed me that she always had great difficulty; that the labors were much protracted; one of her labors (as she expressed it) being dry; that her children were always large; that she labored from two to three days, and expressed herself as very thankful, that she was to have a speedy and, as she hoped, an easy labor. At about one o'clock complete dilatation of the os had taken place, and rupturing the membranes, the head passed into the pelvic cavity. In a short time I discovered that the head had become impacted, and she soon began suffering severe neuralgic pains along the course of the sciatic nerve, followed by severe muscular contractions of the right limb. I informed her of what had taken place and what was required. As I had unfortunately left my forceps at home, I immediately despatched a messenger for them, that I might have them as soon as possible, if I did not succeed in overcoming the impaction. During the intervals of uterine contractions, I succeeded in pressing the head up and out of the pelvic cavity, and supporting the anterior portion of the head until the next contraction, I allowed the vertex to dip down into the pelvic cavity. My efforts at overcoming the difficulty were successful, for during the following one or two contractions I believe the head passed the original point of impaction. But during the uterine contractions following, which were exceedingly severe, the head unfortunately became fixed again so firmly that I could not dislodge it. The neuralgic pains and muscular contractions returned much more severely than before. Very fortunately the messenger soon returned with my instruments. Applying my forceps (Hodge's) immediately, I delivered without delay a 12½ lb. male child. The pains and muscular contractions in the limbs were immediately relieved, followed by numbness through the entire limb and paralysis of that limb which remained some four or five months, but finally gave way under treatment of electricity and friction to the limb and sulphate of quinine and strychnia internally. Otherwise she made a good recovery, and was sitting up on the 10th day as was her custom.

The labor was completed at about four o'clock a.m., about four hours after my arrival.

E. A. HALL, M.D.

ODEBOLT, IOWA.

